# Cognitive and functional deficits are associated with white matter abnormalities in two independent cohorts of patients with schizophrenia

**DOI:** 10.1007/s00406-021-01363-8

**Published:** 2021-12-21

**Authors:** Shinichi Yamada, Shun Takahashi, Berend Malchow, Irina Papazova, Sophia Stöcklein, Birgit Ertl-Wagner, Boris Papazov, Ulrike Kumpf, Thomas Wobrock, Katriona Keller-Varady, Alkomiet Hasan, Peter Falkai, Elias Wagner, Florian J. Raabe, Daniel Keeser

**Affiliations:** 1 Department of Psychiatry and Psychotherapy, University Hospital, LMU Munich, Munich, Germany; 2 Department of Neuropsychiatry, Wakayama Medical University, Wakayama, Japan; 3 Clinical Research and Education Center, Asakayama General Hospital, Sakai, Japan; 4 Department of Psychiatry and Psychotherapy, University Medical Center Göttingen, Göttingen, Germany; 5 Department of Psychiatry Psychotherapy and Psychosomatics, Medical Faculty, University of Augsburg, Augsburg, Germany; 6 Department of Radiology, University Hospital, LMU Munich, Munich, Germany; 7 Division of Neuroradiology, Department of Diagnostic Imaging, The Hospital for Sick Children, Toronto, Canada; 8 Department of Psychiatry and Psychotherapy, County Hospitals Darmstadt-Dieburg, Gross-Umstadt, Germany; 9 Institute of Sports Medicine, Hannover Medical School, Hannover, Germany; 10 International Max Planck Research School for Translational Psychiatry (IMPRS-TP), Munich, Germany; 11 NeuroImaging Core Unit Munich (NICUM), University Hospital, LMU Munich, Munich, Germany

**Keywords:** Cognitive deficits, Fractional anisotropy, Global functioning, Schizophrenia, Replication, Confirmation

## Abstract

**Background:**

Significant evidence links white matter (WM) microstructural abnormalities to cognitive impairment in schizophrenia (SZ), but the relationship of these abnormalities with functional outcome remains unclear.

**Methods:**

In two independent cohorts (C1, C2), patients with SZ were divided into two subgroups: patients with higher cognitive performance (SZ-HCP-C1, *n* = 25; SZ-HCP-C2, *n* = 24) and patients with lower cognitive performance (SZ-LCP-C1, *n* = 25; SZ-LCP-C2, *n* = 24). Healthy controls (HC) were included in both cohorts (HC-C1, *n* = 52; HC-C2, *n* = 27). We compared fractional anisotropy (FA) of the whole-brain WM skeleton between the three groups (SZ-LCP, SZ-HCP, HC) by a whole-brain exploratory approach and an atlas-defined WM regions-of-interest approach via tract-based spatial statistics. In addition, we explored whether FA values were associated with Global Assessment of Functioning (GAF) scores in the SZ groups.

**Results:**

In both cohorts, mean FA values of whole-brain WM skeleton were significantly lower in the SCZ-LCP group than in the SCZ-HCP group. Whereas in C1 the FA of the frontal part of the left inferior fronto-occipital fasciculus (IFOF) was positively correlated with GAF score, in C2 the FA of the temporal part of the left IFOF was positively correlated with GAF score.

**Conclusions:**

We provide robust evidence for WM microstructural abnormalities in SZ. These abnormalities are more prominent in patients with low cognitive performance and are associated with the level of functioning.

**Supplementary Information:**

The online version contains supplementary material available at 10.1007/s00406-021-01363-8.

## Introduction

Schizophrenia (SZ) is a severe neuropsychiatric disorder that is associated with poor social [1–3] and occupational functioning [3, 4]. This poor functioning is related to neurocognitive impairment [5–8]. Neurocognitive impairments can be present from the at-risk period to the chronic stages of SZ [9], but not all patients are affected by cognitive deficits. The fifth edition of *Diagnostic and Statistical Manual of Mental Disorders (DSM-V)* describes a broad range of severities of cognitive impairment in SZ, ranging from intact to severe [10]. However, the question remains unanswered whether the different levels of impairment correlate with different neurobiological characteristics. Understanding the relationship between the degree of cognitive impairment and the underlying neurobiology is key in developing innovative neural targets to improve functional outcomes in SZ.

The disconnection hypothesis in SZ has been put forward in the form of various disconnection theories, such as disconnection of fronto-temporal regions [11, 12], formation of cortico-thalamo-cerebellar loops [13], and crossing of interhemispheric fibers in the corpus callosum [14]. White matter (WM) fibers connect brain regions structurally and functionally [15, 16], including the cortices associated with various cognitive domains [17, 18]. WM microstructural abnormalities can be revealed by diffusion tensor imaging (DTI) because the DTI signal is sensitive to the movement of water molecules and WM microstructures were shown to represent directional information of water molecules [19–21] in postmortem animal studies [22–24] and human brain dissections [25–27]. Recently, the ENIGMA Schizophrenia DTI Working Group conducted the largest international multicenter study to date; it included 4322 patients with SZ from 29 independent international cohorts and highlighted a disturbed WM integrity in widespread regions [28]. Previous studies have supported the importance of WM integrity decline for cognitive impairment [29–31] and functioning in SZ [3, 32]. However, to our knowledge, the relationship between WM integrity, cognitive function, and global functioning in SZ has not yet been investigated with the same analytical methods and replicated in two independent samples with different MRI parameters.

Therefore, we performed a replication study that applied a whole-brain exploratory approach and atlas-defined WM regions-of-interest approach via tract-based spatial statistics (TBSS) in two different cohorts. In the present study, we aimed to robustly identify microstructural differences in WM in patients with SZ subdivided into groups with good and poor cognitive performance. Moreover, we aimed to investigate the relationship between regions-of-interest (ROIs) selected on the basis of exploratory findings and functional outcomes in SZ. We hypothesized that brain WM microstructure in SZ is more severely disturbed in patients with poor cognitive performance and poor functioning.

## Methods

### Participants

Participants in cohort (1) were recruited from the University Hospital, LMU Munich, Germany. This study was approved by the local ethics committee of the University Hospital, LMU Munich (project number: 17–13), and written informed consent was obtained from all participants. The patients were diagnosed by two independent, experienced psychiatrists using the criteria of the *International Statistical Classification of Diseases and Related Health Problems, 10th revision (ICD-10)* [33]. Individuals with no current or past mental illness (according to the MINI Plus Interview [34]) were recruited into the HC group. Most patients were treated with antipsychotic medication, daily dosage of antipsychotic was calculated using the chlorpromazine equivalent method [35] and additional treatment with antidepressants and/or a benzodiazepines was assessed. In all patients, symptom severity was determined with the Positive and Negative Syndrome Scale (PANSS) [36] and level of functioning with the Global Assessment of Functioning (GAF) [37]. GAF scores were demeaned (subtracting the group mean from the individual scores, thus, the new mean is zero) to avoid overestimated associations. Parts of cohort (1) individuals were included in previous studies [38–40]. Participants from cohort (2) were recruited at the Department of Psychiatry and Psychotherapy of the University Medical Center Göttingen, and were assessed with the baseline in a previous longitudinal study [41, 42].

The demographic and clinical characteristics of the study participants in cohorts (1) and (2) are shown in Table 1. Each cohort consisted of patients with SZ and HC. In each cohort, the participants with SZ were evenly divided into a group with higher cognitive performance (HCP) and one with lower cognitive performance (LCP) on the basis of the cognitive composite score, as described in below. Cohort (1) comprised 52 HC and 50 patients with SZ, evenly subdivided into an HCP group (SZ-HCP-C1 group, *n* = 25) and LCP group (SZ-LCP-C1 group; *n* = 25), and cohort (2) comprised 27 HC and 48 patients with SZ subdivided into an HCP group (SZ-HCP-C2 group, *n* = 24) and LCP group (SZ-LCP-C2 group, *n* = 24).

**Table 1:** Demographic and clinical characteristics in cohort (1) and (2)

	Cohort (1)										Post hoc comparisons (HC vs SZ-HCP-C1) (HC vs SZ-LCP-C1) (SZ-HCP-C1 vs SZ-LCP-C1)	Cohort (2)										Post hoc comparisons (HC vs SZ-HCP-C2) (HC vs SZ-LCP-C2) (SZ-HCP-C2 vs SZ-LCP-C2)
	HC (*n* = 52)		SZ (*n* = 50)		*p*	SZ-HCP-C1 (*n* = 25)		SZ-LCP-C1 (*n* = 25)		*p*		HC (*n* = 27)		SZ (*n* = 48)		*p*	SZ-HCP-C2 (*n* = 24)		SZ-LCP-C2 (*n* = 24)		*p*	
Age, y, mean (sd)	32.07	10.95	35.16	11.26	0.164^a^	32.56	9.197	37.76	12.66	0.097^b^		36.62	10.91	35.2	12.4	0.642^a^	31.91	10.65	38.66	13.30	0.127^b^	
Sex, *n*, male/female	39/13		42/8		0.261^c^	20/5		22/3		0.416^c^		18/9		33/15		0.852^c^	16/8		17/7		0.307^c^	
Hand preference, *n*, right/left	46/6		46/4		0.547^c^	24/1		22/3		0.531^c^		21/6		42/6		0.270^c^	21/3		21/3		0.544^c^	
Duration of school education, y, mean (sd)	12.27	1.269	11.52	2.187	0.036*^a^	11.76	1.942	11.28	2.424	0.068^b^	(0.702) (0.070) (1.000)	11.96	1.372	11.66		0.425^a^	12.58	1.442	10.75	1.648	< 0.001^b^	(0.424) (0.015*) (< 0.001***)
Duration of illness, y, mean (sd)			8.920	9.023		7.600	7.852	10.24	10.04	0.306^a^				9.916	8.867		7.791	8.005	12.04	9.336	0.097^a^	
PANSS positive score, mean (sd)			13.96	5.409		13.92	5.015	14.00	5.880	0.959^a^				14.33	6.302		13.75	5.712	14.91	6.915	0.527^a^	
PANSS negative score, mean (sd)			16.98	5.235		15.00	4.627	18.96	5.135	0.006**^a^				20.16	9.198		19.33	8.297	21.00	10.12	0.536^a^	
PANSS general score, mean (sd)			30.44	8.435		19.32	7.695	31.56	9.133	0.353^a^				37.33	16.76		33.04	14.49	41.62	18.04	0.076^a^	
PANSS total score, mean (sd)			61.38	16.67		58.24	15.46	64.52	17.55	0.186^a^				71.83	29.98		66.12	25.8	77.54	*33.21*	0.190^a^	
Demean GAF, mean (sd)			55.77	9.66		58.38	9.588	53.16	9.186	0.055^a^				59.93	11.93		61.75	12.42	58.12	*11.38*	0.298^a^	
STM score, *z* scores, mean (sd)	0.516	0.915	− 0.537	0.794	< 0.001^a^	− 0.067	0.691	− 1.007	0.592	< 0.001^b^	(0.010**) (< 0.001***) (< 0.001***)	0.276	0.873	− 0.155	1.051	0.074^a^	0.440	0.942	− 0.751	0.792	< 0.001^b^	(1.000) (< 0.001***) (< 0.001***)
LMT score, *z* scores, mean (sd)	0.543	0.667	− 0.564	0.989	< 0.001^a^	0.142	0.604	− 1.271	0.727	< 0.001^b^	(0.052) (< 0.001***) (< 0.001***)	0.234	0.857	− 0.132	1.067	0.131^a^	0.485	0.746	− 0.749	0.988	< 0.001^b^	(0.924) (< 0.001***) (< 0.001***)
TMT-A, time (s), *z* scores, mean (sd)	− 0.467	0.858	0.488	0.914	< 0.001^a^	0.013	0.791	0.963	0.781	< 0.001^b^	(0.054) (< 0.001***) (< 0.001***)	− 0.267	0.632	0.150	1.144	0.045*^a^	− 0.578	0.739	0.879	1.011	< 0.001^b^	(0.518) (< 0.001***) (< 0.001***)
TMT-B, time (s), *z* scores, mean (sd)	− 0.420	0.498	0.437	1.199	< 0.001^a^	− 0.244	0.487	1.119	1.315	< 0.001^b^	(1.000) (< 0.001***) (< 0.001***)	− 0.293	0.965	0.165	1.001	0.058^a^	− 0.466	0.503	0.797	0.981	< 0.001^b^	(1.000) (< 0.001***) (< 0.001***)
Cognitive composite score, *z* scores, mean (sd)	0.487	0.548	− 0.507	0.760	< 0.001^a^	0.076	0.375	− 1.090	0.574	< 0.001^b^	(0.005**) (< 0.001***) (< 0.001***)	0.268	0.565	− 0.150	0.826	0.022*^a^	0.492	0.471	− 0.794	0.557	< 0.001^b^	(0.417) (< 0.001***) (< 0.001***)
Daily dose of antipsychotics, CPZ equivalents, mean (sd)			472.1	325.6		437.4	265.2	506.8	379.0	0.456^a^				548.9	545.4		600.0	646.3	497.8	429.9	0.522^a^	
Antidepressant, *n*, with/without			8/42			3/22		5/20		0.440^c^				11/37			5/19		6/18		0.731^c^	
Benzodiazepine, *n*, with/without			7/43			2/23		5/20		0.221^c^				6/41			1/23		5/19		0.080^c^	

Significant differences among the three groups are marked. Values marked with ***, ** and * are significant at *p* < 0.001, *p* < 0.01, and *p* < 0.05, respectively

*HC* healthy controls, *SZ* schizophrenia, *HCP* high cognitive performer, *LCP* low cognitive performer, *SD* standard deviation, *PANSS* Positive and Negative Syndrome Scale, *GAF* Global Assessment of Functioning, *CPZ* *equivalents* chlorpromazine equivalents

^a^Independent samples *t* test

^b^Analysis of variance and Bonferroni’s post hoc test

^c^Chi-square test

### Neuropsychological measurements in cohorts (1) and (2)

We used neurocognitive instruments that are related to functional deficits in SZ [7, 30, 43]. Neurocognitive function was assessed by experienced psychologists with the short-term memory (STM) and long-term memory (LTM), which were obtained from previous factor analyses of the Verbal Learning Memory Test [44], and the Trail Making Test parts A (TMT-A) and B (TMT-B) [45]. Z scores, that consisted of number of remembered words (STM, LTM, high score = good performance), and seconds to complete task (TMT-A, TMT-B, high score = poor performance), were used for the neuropsychological tests. Within each cohort, a cognitive composite score was calculated with the following equation: {(STM z scores) + (LTM z scores) + (− 1) × (TMT-A z scores) + (− 1) × (TMT-B z scores)}/4.

### Magnetic resonance imaging data acquisition and DTI parameters in cohort (1)

All magnetic resonance imaging (MRI) examinations were performed with a 3.0 T MR scanner (Magnetom Skyra, Siemens Healthcare, Erlangen, Germany) with a standard 20-channel phased-array head coil. DTI was performed with 64 non-collinear diffusion-encoding directions and the following parameters: repetition time, 9600 ms; echo time, 95 ms; field of view, 244 mm; voxel size, 2.0 × 2.0 × 2.0 mm; slice thickness, 2.0 mm; 65 slices; and multiple diffusion weighting *b* values (*b* = 1000 s/mm^2^ and *b* = 0).

### MRI data acquisition and DTI parameter in cohort (2)

All MRI examinations were performed with a 3.0 T MR scanner (Magnetom TIM Trio, Siemens Healthcare, Erlangen, Germany) with a standard 8-channel phased-array head coil. DTI was performed with 12 non-collinear diffusion-encoding directions and the following parameters: repetition time, 6500 ms; echo time, 96 ms; field of view, 256 mm; voxel size, 2.0 × 2.0 × 2.0 mm; slice thickness, 2.0 mm; 49 slices; and multiple diffusion weighting *b* values (*b* = 1000 s/mm^2^ and *b* = 0).

### Imaging analysis in cohorts (1) and (2)

DTI data were processed with TBSS programs [46] in the FMRIB software Library (FSL), version 6.0.0. [47]. The Brain Extraction Tool was used to create a binary mask from the non-diffusion-weighted data, and the diffusion tensor and associated parameters such as fractional anisotropy (FA) maps were calculated with the DTIFIT program implemented in the FSL. Nonlinear transformation and affine registration were performed to normalize all FA data into a standard space with the nonlinear registration tool FNIRT [48]. Normalized FA images were averaged to create a mean FA image, and a mean FA skeleton was created by taking the centers of all tracts common to all participants. The voxel values of each participant’s FA map were projected onto the skeleton by searching the local maxima along the perpendicular direction from the skeleton. The resulting data were fed into the voxel-wise statistical analysis described in Sect. 2.5.

### Statistical analyses in cohorts (1) and (2)

In each cohort, differences in demographic and clinical characteristics between the HC and SZ groups were analyzed by independent samples *t* tests for continuous variables (age, duration of school education, the *z* score of four neurocognitive tests and the cognitive composite score) and chi-square tests for categorical variables (sex distribution and hand preference), with a significance level of *α* < 0.05. Differences in demographic and clinical characteristics between the SZ-HCP and SZ-LCP groups in each cohort were analyzed with independent samples *t* tests for continuous variables (duration of illness, PANSS subscores, GAF scores, chlorpromazine daily dose equivalents) and chi-square tests for the number of patients taking medication, with a significance level of *α* < 0.05. Differences in demographic and clinical characteristics between the HC, SZ-HCP, and SZ-LCP groups in each cohort were analyzed by analysis of variance and Bonferroni’s post hoc test for continuous variables (age, duration of school education, and the *z* scores of each neurocognitive test) and chi-square tests for categorical variables (sex distribution and hand preference), with a significance level of *α* < 0.05. Group differences in demographic and clinical characteristics between the SZ groups in cohorts (1) and (2) were analyzed by independent samples *t* tests for continuous variables (age and duration of school education) and chi-square tests for categorical variables (sex distribution and hand preference), with a significance level of *α* < 0.05. All statistical analyses were performed with IBM SPSS statistics 20.

#### Whole-brain exploratory approach

Voxel-wise statistics of the skeletonized FA data were applied using randomize in FSL, version 6.0.0. The HC and SZ groups were compared by an analysis of covariance design, with age and sex as nuisance covariates. We randomly performed permutation-based testing with 5000 permutations and inference by threshold-free cluster enhancement (TFCE) with a threshold of less than 0.05. The mean FA values of the whole skeleton in the HC, SZ-HCP, and SZ-LCP groups were examined for differences by analysis of variance and Bonferroni’s post hoc test, with age and sex as covariates, with a significance level of *α* < 0.05.

#### Atlas-defined WM regions-of-interest approach

For atlas-based segmentation, all extracted skeletons were overlaid with the Johns Hopkins University DTI-based WM Atlas in FSL [49, 50]. Differences of the mean FA values in 20 ROIs in the HC and SZ groups were examined by independent samples *t* tests, with age and sex as covariates, with significance set at *p* < 0.00125. (= 0.05/40 WM tracts because 20 WM tracts were examined in each cohort). The mean FA values of 20 ROIs in the HC, SZ-HCP, and SZ-LCP groups were examined for differences by analysis of variance and Bonferroni’s post hoc test, with age and sex as covariates and significance set at *p* < 0.00125 (= 0.05/40 WM tracts because 20 WM tracts were examined in each cohort). In the voxels with a statistical difference in the mean FA values of 20 ROIs, voxel-wise multiple regression analyses were performed with TBSS to examine the relationship between FA values and demean GAF scores. We used 5000 permutations to calculate FA values using age and sex as covariates. Spearman's rank correlation test was carried out between the demeaned GAF scores and mean FA values of the voxels that were statistically significant in the voxel-wise multiple regression analysis.

## Results

### Demographic and clinical characteristics in cohorts (1) and (2)

Table 1 illustrates demographic and clinical characteristics of both cohorts. In cohort (1), no differences were observed in age, sex, or hand preference between the HC and SZ groups, however, duration of school education was significantly lower in the SZ group than in the HC group. Age, sex, hand preference, and duration of school education were not different between the HC, SZ-HCP, and SZ-LCP groups. Furthermore, duration of illness, PANSS positive, general, and total scores, GAF scores, chlorpromazine daily dose equivalents, and the number of patients taking an antidepressant or benzodiazepine were not different between the SZ-HCP and SZ-LCP groups. However, the SZ-LCP group had significantly higher PANSS negative scores than the SZ-HCP group (*p* < 0.01).

In cohort (2), no differences in age, sex, hand preference, or duration of school education were observed between the HC and SZ groups. Age, sex, and hand preference were not different between the HC, SZ-HCP, and SZ-LCP groups, but duration of school education was significantly lower in the SZ-LCP group than in the SZ-HCP group (*p* < 0.001). No significant differences were found between the SZ-HCP and SZ-LCP groups with regard to the duration of illness, PANSS subscales, GAF scores, chlorpromazine daily dose equivalents, and the number of patients taking an antidepressant or benzodiazepine.

Further analyses of demographic and clinical characteristics between the SZ groups in cohorts (1) and (2) are shown in Supplementary Table 1. The SZ group in cohort (2) had significantly higher PANSS negative, general, and total scores than the SZ group in cohort (1) (*p* < 0.05).

### Group comparison of FA in cohorts (1) and (2)

#### Whole-brain exploratory approach

**Fig. 1:**
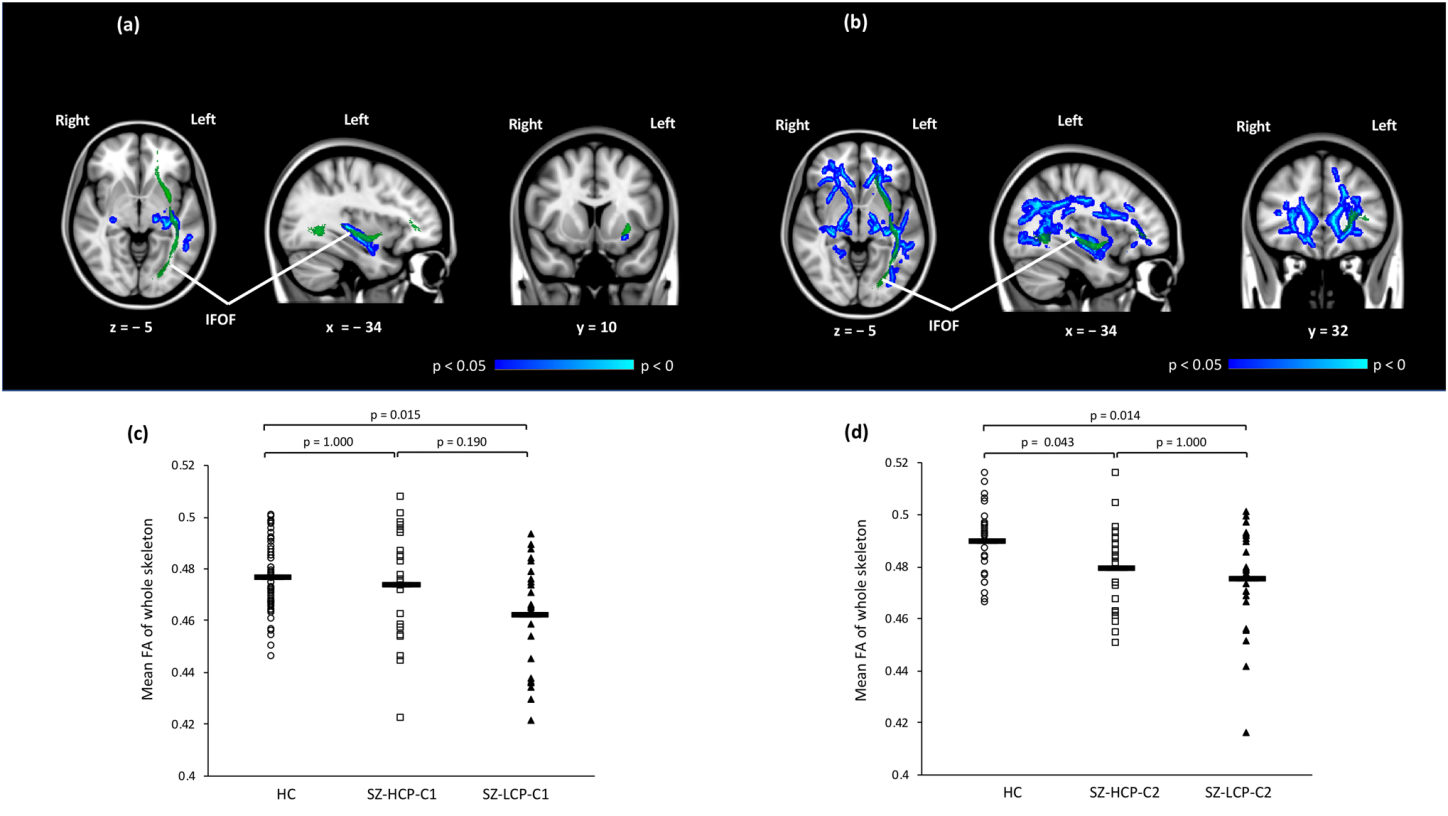
**a** Difference in fractional anisotropy (FA) values between the healthy controls (HC) and schizophrenia (SZ) groups in cohort (1). Blue to light blue voxels indicate regions where the FA values were significantly lower in the SZ group than in the HC group (*p* < 0.05). **b** Difference in FA values between the HC and SZ groups in cohort (2). Blue to light blue voxels indicate regions where the FA values were significantly lower in the SZ group than in the HC group (*p* < 0.05). **c** Differences in mean fractional anisotropy (FA) values of whole-brain white matter (WM) skeleton in healthy controls (HC) and in patients with schizophrenia with higher cognitive performance (SZ-HCP-C1) and lower cognitive performance (SZ-LCP-C1) in cohort (1). The circles represent mean FA values of the whole skeleton in the HC group; the squares represent mean FA values of the whole skeleton in the SZ-HCP-C1 group; and the triangles represent mean FA values of the whole skeleton in the SZ-LCP-C1 group. **d** Differences in mean FA values of whole-brain WM skeleton in the HC, SZ-HCP-C2, and SZ-LCP-C2 groups in cohort (2). The circles represent mean FA values of the whole skeleton in the HC group; the squares represent mean FA values of the whole skeleton in the SZ-HCP-C2 group; and the triangles represent mean FA values of the whole skeleton in the SZ-LCP-C2 group. *FA* fractional anisotropy, *HC* healthy controls, *SZ* schizophrenia, *SZ-HCP-C1* patients with schizophrenia and higher cognitive performance in cohort (1), *SZ-HCP-C2* patients with schizophrenia and higher cognitive performance in cohort (2), *SZ-LCP-C1* patients with schizophrenia with lower cognitive performance in cohort (1), *SZ-LCP-C2 *patients with schizophrenia with lower cognitive performance in cohort (2)

In cohort (1), the FA values in the SZ group were significantly lower than those in the HC group in the left temporal basal areas (*p* < 0.05, Fig. 1a). The mean FA of the whole-brain WM skeleton was significantly lower in the SZ-LCP group than in the HC group (*p* < 0.05, Fig. 1c).

In cohort (2), the FA values in the SZ group were significantly lower than those in the HC group in widespread regions (*p* < 0.05, Fig. 1b). The mean FA of the whole-brain WM skeleton was significantly lower in the SZ-HCP and SZ-LCP groups than in the HC group (*p* < 0.05, Fig. 1d).

#### Atlas-defined WM regions-of-interest approach

The results of our analysis of the significant differences in WM skeleton mean FA in patients and HC in the major WM tracts as defined by the Johns Hopkins University WM Atlas are shown in Table 2. In both cohorts, the WM skeleton mean FA values in the SZ and SZ-LCP groups were significantly lower than those in the HC group in the left IFOF (Fig. 1, Table 2), i.e., significant cognition-related FA reductions in SZ were found in the left IFOF.

**Table 2:** Differences in fractional anisotropy in the Johns Hopkins University White-Matter Tractography Atlas-based tract

	Cohort (1)										Post hoc comparison (HC vs SZ-HCP-C1) (HC vs SZ-LCP-C1) (SZ-HCP-C1 vs SZ-LCP-C1)	Cohort (2)										Post hoc comparisons (HC vs SZ-HCP-C2) (HC vs SZ-LCP-C2) (SZ-HCP-C2 vs SZ-LCP-C2)
	HC		SZ		*p*	SZ-HCP-C1		SZ-LCP-C1		*p*		HC		SZ		*p*	SZ2-HCP-C2		SZ-LCP-C2		*p*	
Left anterior thalamic radiation, mean (sd)	0.482	0.015	0.472	0.019	0.005*	0.475	0.020	0.486	0.017	0.013*	(0.154) (0.013*) (1.000)	0.449	0.014	0.442	0.025	0.083	0.449	0.025	0.435	0.025	0.013*	(1.000) (0.015*) (0.054)
Right anterior thalamic radiation, mean (sd)	0.471	0.018	0.461	0.018	0.031*	0.463	0.019	0.459	0.018	0.072		0.437	0.015	0.431	0.024	0.155	0.436	0.026	0.427	0.023	0.117	
Left cingulum cingulate gyrus, mean (sd)	0.634	0.031	0.618	0.032	0.046*	0.628	0.028	0.609	0.034	0.020*	(1.000) (0.019*) (0.147)	0.609	0.029	0.603	0.041	0.394	0.612	0.039	0.594	0.042	0.143	
Right cingulum cingulate gyrus, mean (sd)	0.592	0.032	0.574	0.044	0.067	0.579	0.040	0.570	0.048	0.168		0.566	0.037	0.561	0.043	0.438	0.566	0.041	0.555	0.046	0.517	
Left cingulum hippocampus, mean (sd)	0.559	0.044	0.549	0.063	0.554	0.561	0.061	0.537	0.064	0.087		0.509	0.049	0.489	0.049	0.020*	0.482	0.057	0.496	0.039	0.043*	(0.043*) (0.551) (1.000)
Right cingulum hippocampus, mean (sd)	0.562	0.062	0.554	0.063	0.735	0.555	0.079	0.554	0.061	0.670		0.502	0.050	0.493	0.049	0.178	0.492	0.052	0.493	0.047	0.400	
Left corticospinal tract, mean (sd)	0.637	0.020	0.630	0.020	0.100	0.631	0.022	0.628	0.019	0.253		0.585	0.025	0.582	0.028	0.790	0.590	0.026	0.574	0.028	0.195	
Right corticospinal tract, mean (sd)	0.637	0.021	0.632	0.025	0.369	0.632	0.029	0.633	0.022	0.575		0.602	0.026	0.593	0.031	0.309	0.598	0.029	0.588	0.032	0.481	
Forceps major, mean (sd)	0.706	0.019	0.694	0.031	0.094	0.694	0.031	0.693	0.032	0.247		0.692	0.022	0.679	0.027	0.034*	0.685	0.028	0.674	0.025	0.057	
Forceps minor, mean (sd)	0.584	0.023	0.567	0.027	< 0.001***	0.574	0.028	0.56	0.025	0.002**	(0.033*) (0.003**) (1.000)	0.577	0.029	0.566	0.028	0.120	0.577	0.027	0.554	0.025	0.017*	(1.000) (0.021*) (0.049*)
Left inferior fronto-occipital fascicles, mean (sd)	0.560	0.023	0.534	0.023	< 0.001***	0.537	0.021	0.531	0.025	< 0.001***	(0.002**) (< 0.001***) (1.000)	0.530	0.021	0.517	0.025	0.014*	0.528	0.025	0.507	0.019	< 0.001***	(1.000) (< 0.001***) (0.007**)
Right inferior fronto-occipital fascicles, mean (sd)	0.551	0.023	0.540	0.023	0.055	0.545	0.018	0.536	0.027	0.102		0.525	0.021	0.516	0.028	0.112	0.528	0.027	0.503	0.022	< 0.001***	(1.000) (0.002**) (0.002**)
Left inferior longitudinal fascicles, mean (sd)	0.555	0.023	0.533	0.027	< 0.001***	0.536	0.024	0.531	0.030	0.004**	(0.020*) (0.008**) (1.000)	0.515	0.025	0.504	0.024	0.053	0.512	0.024	0.495	0.02	0.020*	(1.000) (0.017**) (0.130)
Right inferior longitudinal fascicles, mean (sd)	0.573	0.023	0.563	0.029	0.115	0.566	0.026	0.560	0.033	0.274		0.524	0.024	0.515	0.026	0.183	0.527	0.024	0.503	0.024	0.006**	(1.000) (0.014*) (0.011*)
Left superior longitudinal fascicles, mean (sd)	0.522	0.023	0.506	0.024	0.006*	0.507	0.026	0.505	0.024	0.023*	(0.044*) (0.076) (1.000)	0.497	0.025	0.487	0.025	0.097	0.490	0.026	0.484	0.024	0.245	
Right superior longitudinal fascicles, mean (sd)	0.522	0.025	0.511	0.025	0.100	0.507	0.027	0.516	0.023	0.102		0.512	0.027	0.503	0.028	0.158	0.509	0.028	0.497	0.027	0.183	
Left superior longitudinal fascicles, temporal part, mean (sd)	0.568	0.027	0.553	0.027	0.023	0.552	0.027	0.553	0.027	0.068		0.536	0.029	0.527	0.029	0.211	0.527	0.032	0.527	0.026	0.420	
Right superior longitudinal fascicles, temporal part, mean (sd)	0.560	0.031	0.553	0.028	0.290	0.549	0.027	0.558	0.028	0.256		0.541	0.031	0.534	0.028	0.305	0.538	0.031	0.529	0.025	0.445	
Left uncinate fascicles, mean (sd)	0.549	0.036	0.521	0.032	< 0.001***	0.526	0.033	0.516	0.029	0.003**	(0.038*) (0.004**) (1.000)	0.487	0.028	0.481	0.035	0.239	0.487	0.034	0.476	0.035	0.209	
Right uncinate fascicles, mean (sd)	0.582	0.033	0.562	0.038	0.027	0.562	0.038	0.561	0.039	0.084		0.524	0.035	0.513	0.039	0.125	0.515	0.042	0.511	0.037	0.286	

One-way ANOVA and Bonferroni’s post hoc test with age and sex as covariates. Significant differences among the three groups are marked. Values marked with ***, ** and * are significant at *p* < 0.05/40 = 0.00125, *p* < 0.01 and *p* < 0.05, respectively

**Fig. 2:**
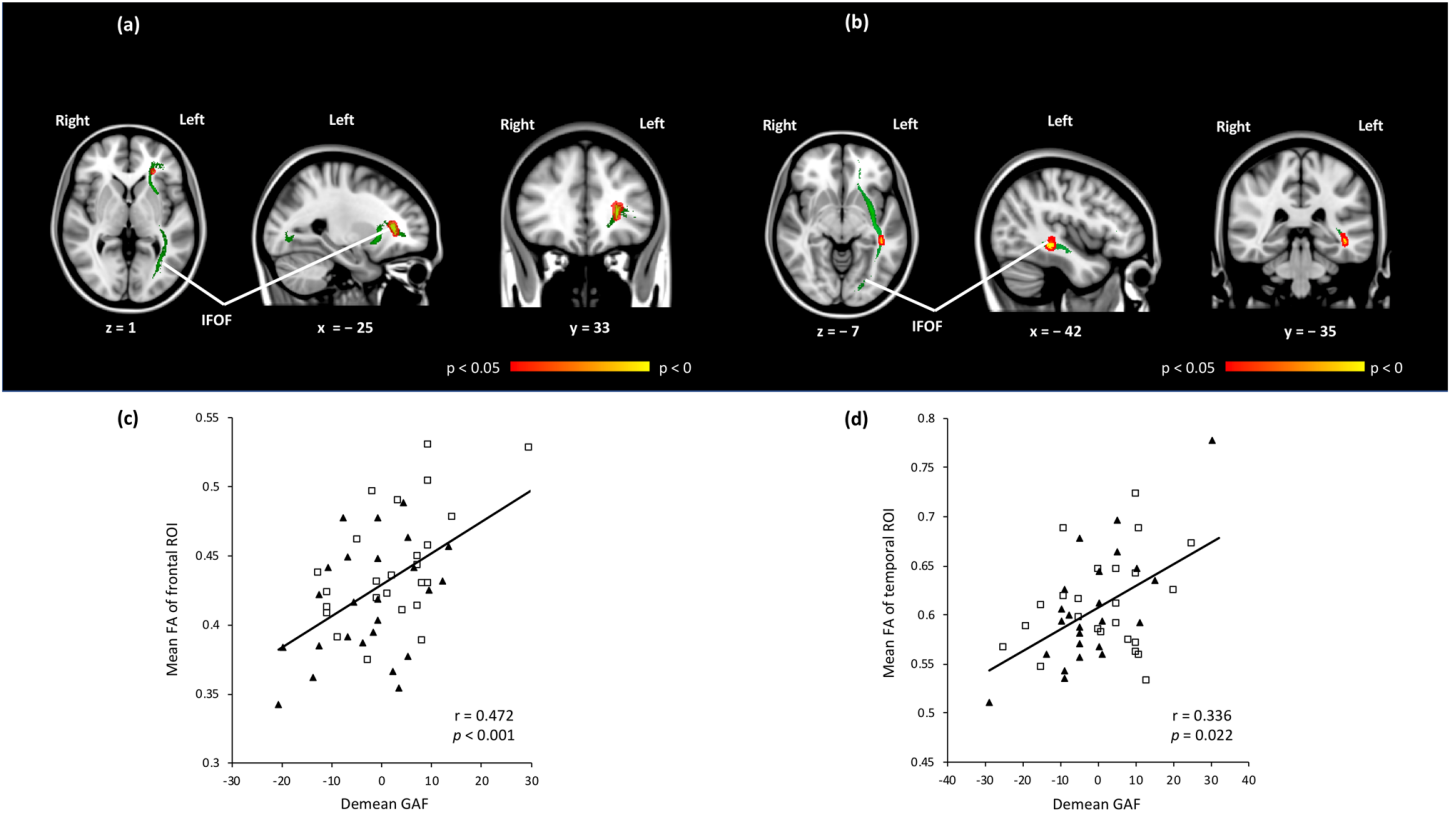
**a** Red-yellow voxels indicate a significant positive relation between the fractional anisotropy (FA) values in the left inferior fronto-occipital fasciculus (IFOF) and the demean Global Assessment of Functioning (GAF) scores in the schizophrenia (SZ) group in cohort (1). **b** Red-yellow voxels indicate a significant positive relation between the FA values in the left IFOF and the demean GAF scores in the SZ group in cohort (2). **c** Scatter plot showing the relation of the demean Global Assessment of Functioning (GAF) scores and mean fractional anisotropy (FA) values of the voxels that showed a statistically significant correlation in voxel-wise multiple regression analysis in **a**. The squares represent patients with schizophrenia and higher cognitive performance in cohort (1), and the triangles represent the group of patients with schizophrenia and lower cognitive performance in cohort (1). **d** Scatter plot showing the relation of the demean GAF scores and mean FA values of the voxels that reached a statistically significant correlation in voxel-wise multiple regression analysis in **b**. The squares represent patients with schizophrenia and higher cognitive performance in cohort (2), and the triangles represent patients with schizophrenia and lower cognitive performance in cohort (2). *FA* fractional anisotropy, *GAF* global assessment of functioning, *IFOF* inferior front-occipital fascicles, *SZ* schizophrenia, *ROI* region of interest

Finally, multiple regression analysis revealed that in cohort (1), FA values in the frontal part of the left IFOF were significantly associated with the demean GAF scores (r = 0.472, *p* < 0.001, Fig. 2a,c); and in cohort (2), FA values in the temporal part of the left IFOF were significantly associated with the demean GAF scores (*r* = 0.336, *p* = 0.022, Fig. 2b,d), i.e., significant positive associations were found between the cognitive-related FA and the demean GAF scores in the fronto-temporal part of the left IFOF in SZ.

## Discussion

Our results provide robust evidence of WM microstructural abnormalities in patients with SZ, especially in those patients with lower cognitive performance (SZ-LCP). Second, in the SZ group we found a significant positive relationship between cognition-related FA values in the fronto-temporal part of the left IFOF and the GAF score.

Our results are consistent with previous findings which revealed that WM volumes were significantly smaller in patients with SZ with cognitive impairment than in healthy individuals but WM volumes in patients without cognitive impairment were not [51]. Moreover, Pérez-Iglesias et al. [52] reported that patients with SZ with cognitive impairment showed a significantly greater decrease in FA values than patients without cognitive impairment.

In this study, we could replicate in two independent cohorts of patients with SZ, that especially patients with poor cognitive performance were affected by WM microstructural abnormalities. Moreover, cognition-related WM abnormalities, which were mainly found in fronto-temporal parts of the left IFOF, were related to general, social, and occupational functioning.

It is worth emphasizing here that in our study FA was measured with different MRI scan parameters in two independent cohorts. A clinical application of structural MRI in clinical trials requires a certain robustness of readouts across different scanners and protocols, despite all biological and technical variability.

The advantage of the current study is the replication of our findings in two independent cohorts and thus increases generalizability. Therefore, our results provide robust evidence that emphasizes the relevance of large reductions in FA as indicating a neurobiological mechanism of SZ in patients with severe cognitive impairment. Beyond the group comparison, the future goal is to find a framework for individual patients [53] to allow MRI images from individuals to be directly compared with a reference. In particular, the increase in the use of MRI-based outcome measurements in clinical trials [54] requires a more precise definition and standardization, especially considering assessments of individuals.

In its atlas-based analysis, the current study found a significant positive relationship in both cohorts between cognition-related FA, mainly in the fronto-temporal part of the left IFOF, and demean GAF scores in patients. The IFOF is the longest associative bundle and connects the occipital cortex, superior parietal lobule, and temporal basal areas to the frontal lobe [55, 56]. Moreover, the fronto-temporal part of the left IFOF is the region of greater interest in SZ: As early as the last century, Wernicke [57] and Kraepelin [58] suggested the importance of the fronto-temporal network in the neuropathology of SZ, and later brain investigations with various neuroimaging methods reinforced this idea [12, 59, 60]. Previous DTI findings implicated prefrontal and temporal lobes [61–63] and the fiber tracts connecting these regions [64] in SZ. Our current finding of an association of the anisotropic reductions in the left IFOF with the cognitive composite and reduced GAF scores may be seen as confirming some previous reports from other DTI studies in SZ that used other cognitive test batteries [31, 65]. In summary, this replication study in a cohort from our previous studies [41, 42] and a new cohort extends previous work [31, 65]. We demonstrated that the origin of neurocognitive deficits and poor functional outcomes in patients with SZ may be related to neurobiological abnormalities in the left IFOF of brain networks in the temporal and frontal cortex.

An interesting finding from our analysis of the whole skeleton is that the FA values in widespread regions were significantly lower in the SZ group than in the HC group in cohort (2) but not in cohort (1). Another interesting finding from our atlas-based analysis is that we found a significant positive relation between the FA values and the demean GAF scores in the temporal part of the left IFOF in cohort (2) but in the frontal part of the left IFOF in cohort (1), i.e., the local regions in the left IFOF could not be replicated in both cohorts. This discrepancy between the independent cohorts might be explained, at least in part, by some factors affecting FA, such as differences in symptom severity for SZ [28], the use of different channel head coils in MRI research [66] and other dimensions (e.g. aggression or impulsivity[67]) that were not included in the present study.

Some limitations of this study must be noted. First, the patients were taking a variety of pharmacological agents not only at the time of scanning, but prior to the scanning (i.e., lifetime use of medication). Second, they were evenly divided with regard to the cognitive composite score and could not be classified according to unique score criteria (e.g., > 1 SD below the normative mean), though previous findings show that approximately one quarter of schizophrenia have similar cognitive performance as healthy [51]. Future studies are necessary using a greater number of patients, who are classified according to unique score criteria, with limited medication exposure and matched lifetime use of medication to confirm the results of our study.

The main strength of this study is the replication approach that used independent samples with different MRI parameters. The results provide a foundation for developing the neurobiological basis of cognitive function, which could serve as a functional proxy in SZ and other psychiatric disorders. Of particular importance will be the design of an approach to transform individual changes of the structural and functional connectome towards a clinical application. Here, cognition and functionality for psychiatric disorders will also play an important role across diagnoses.

## Supplementary Information

Below is the link to the electronic supplementary material.

Supplementary file1 (DOCX 28 KB)

## Abbreviations

DTI: Diffusion tensor imaging
FA: Fractional anisotropy
FSL: FMRIB software library
HC: Healthy controls
IFOF: Inferior fronto-occipital fasciculus
GAF: Global assessment of functioning
PANSS: Positive and negative syndrome Scale
STM: Short-term memory
SZ: Schizophrenia
SZ-HCP: Schizophrenia with higher cognitive performance
SZ-LCP: Schizophrenia with lower cognitive performance
TBSS: Tract-based spatial statistics
TMT-A: Trail Making Test part A
TMT-B: Trail Making Test part B
LTM: Long-term memory
WM: White matter
