# Supplementary material for: Cognitive and functional deficits are associated with white matter abnormalities in two independent cohorts of patients with schizophrenia
Source: Eur Arch Psychiatry Clin Neurosci. 2021 Dec 21;272(6):957–69. doi: 10.1007/s00406-021-01363-8 (PMC9388472; doi:10.1007/s00406-021-01363-8)
Supplement: Supplementary file 1 — Supplementary file1 (DOCX 28 KB) [file 406_2021_1363_MOESM1_ESM.docx]

# Supplementary Table S1: Demographic and clinical characteristics in cohort (1) and (2)

|  | Cohort (1) | |  | Cohort (2) | |  | |
| --- | --- | --- | --- | --- | --- | --- | --- |
|  | SZ | |  | SZ | |  | *p* |
|  | (n = 50) | |  | (n = 48) | |  |  |
| Age, y, mean (sd) ^a^ | 35.16 | 11.26 |  | 35.2 | 12.40 |  | 0.958 |
| Sex, n, male/female | 42/8 |  |  | 33/15 |  |  | 0.096 |
| Hand preference, n, right/left | 46/4 |  |  | 42/6 |  |  | 0.520 |
| Duration of school education, y, mean (sd) ^a^ | 11.52 | 2.187 |  | 11.66 |  |  | 0.728 |
| Duration of illness, y, mean (sd) ^a^ | 8.920 | 9.023 |  | 9.916 | 8.867 |  | 0.583 |
| PANSS positive score, mean (sd) ^a^ | 13.96 | 5.409 |  | 14.33 | 6.302 |  | 0.753 |
| PANSS negative score, mean (sd) ^a^ | 16.98 | 5.235 |  | 20.16 | 9.198 |  | 0.039* (Cohort1 < Cohort2) |
| PANSS general score, mean (sd) ^a^ | 30.44 | 8.435 |  | 37.33 | 16.76 |  | 0.013* (Cohort1 < Cohort2) |
| PANSS total score, mean (sd) ^a^ | 61.38 | 16.67 |  | 71.83 | 29.98 |  | 0.037* (Cohort1 < Cohort2) |
| GAF, mean (sd) ^a^ | 55.77 | 9.66 |  | 59.93 | 11.93 |  | 0.06 |
| Daily dose of antipsychotics, CPZ equivalents, mean (sd) ^a^ | 472.1 | 325.6 |  | 548.9 | 545.4 |  | 0.403 |
| Antidepressant, n, with/without | 8/42 |  |  | 11/37 |  |  | 0.386 |
| Benzodiazepine, n, with/without | 7/43 |  |  | 6/41 |  |  | 0.858 |

Significant differences between the 2 groups are marked. Values marked with * are significant at p < 0.05.
